# Living Donor Liver Transplantation for Hepatocellular Carcinoma: Appraisal of the United Network for Organ Sharing Modified TNM Staging

**DOI:** 10.3389/fsurg.2020.622170

**Published:** 2021-01-21

**Authors:** Abu Bakar Hafeez Bhatti, Anum Waheed, Nasir Ayub Khan

**Affiliations:** ^1^Division of Hepato-Pancreatico-Biliary Surgery, Shifa International Hospital Islamabad, Islamabad, Pakistan; ^2^Division of Anesthesiology, Shifa International Hospital Islamabad, Islamabad, Pakistan

**Keywords:** hepatocellula carcinoma, liver tranpslant, down staging, recurrence free survival, united network for organ sharing

## Abstract

**Background:** In deceased donor liver transplantation (DDLT), transplant eligibility for T3–T4 HCC requires successful downstaging (DS). Living donor liver transplantation (LDLT) can be considered selectively in these patients without DS, but its role is not defined. The objective of the current study was to assess outcomes of LDLT for HCC based on UNOS staging with no prior DS.

**Materials and Methods:** Patients who underwent LDLT for HCC (*n* = 262) were staged based on modified UNOS TNM staging. High-risk factors were identified and 5-year recurrence free survival was compared in patients with T2–T4 HCC.

**Results:** Median follow-up was 30.2 (16.4–46.3) months. Recurrence rate in T1, T2, T3, T4a, and T4b HCC was 0, 10.1, 16.1, 5.9, and 37.5% (*P* = 0.02), respectively. On multivariate analysis, AFP > 600 ng/mL [HR:11.7, *P* < 0.001] and T4b HCC (macrovascular invasion) [HR = 5.6, *P* = 0.03] were predictors of recurrence. After exclusion of AFP > 600 ng/mL, 5-year RFS for T2, T3, and T4a HCC was 94, 86, and 92% (*P* = 0.3). Rate of microvascular invasion between T2 and T3 HCC was 24.3 vs. 53.6% (*P* = 0.005), and between T2 and T4a HCC was 24.3 vs. 36.7% (*P* = 0.2). Overall, 26 (19.4%) patients were overstaged and 23 (17.1%) were understaged on preoperative imaging. The 5-year RFS in patients with identical preoperative and histopathological staging was 94, 87, and 94% (*P* = 0.6).

**Conclusion:** LDLT without prior DS leads to comparable survival for UNOS T2, T3, and T4a HCC as long as AFP is < 600 ng/mL.

## Introduction

Liver transplantation (LT) is an established treatment modality for patients with liver cirrhosis and hepatocellular carcinoma (HCC) (1). In patients with HCC, Milan criteria and University of California San Francisca criteria (UCSF) remain the benchmark for patient selection (2, 3). These criteria are based on tumor-related features, including tumor size and number. In the United States, patients with united network for organ sharing (UNOS) T2 HCC (Milan criteria) are eligible for MELD exception points for LT. Patients with UNOS T3 HCC are granted exception points if successful downstaging (DS) to T2 HCC is demonstrated with locoregional therapy (LRT). For treatment allocation, alpha fetoprotein (AFP) has also been incorporated, and patients with AFP > 1,000 ng/mL are not granted MELD exception points unless AFP drops below 500 ng/mL with LRT^1^.

Successful DS as evidenced by tumor shrinkage or a drop in AFP serves as a surrogate for good tumor biology, and allows LT to be considered in these patients (4, 5). However, there is lack of well-established criteria for DS, not all patients with advanced HCC (T3–T4) are suitable candidates, and some might decompensate further with potential risk of death with DS (6, 7). There is growing evidence that bio markers like AFP might play an important role in predicting post-transplant recurrence in patients with tumors outside traditional transplant criteria (8–10). Thus, DS might not be needed in all patients with T3–T4 HCC to determine transplant candidacy. With DDLT, upfront LT for T3–T4 HCC remains less probable outside the setting of a clinical trial. As a result, outcomes of LT without prior DS for T3–T4 HCC have not been compared with T2 HCC.

As a high-volume living donor liver transplant (LDLT) center, we have performed a substantial number of transplants for HCC exceeding traditional transplant criteria. This provides a unique opportunity to assess outcomes in patients with HCC who did not receive pre transplant LRT.

The objective of the current study was to assess outcomes in patients who underwent LDLT for early and advanced HCC based on modified UNOS TNM staging.

## Materials and Methods

Between April 2012 and June 2020, 932 LDLTs were performed at our center. All adult patients with a preoperative diagnosis of HCC based on imaging who underwent LDLT between April 2012 and September 2019 were reviewed. A total of 254 patients underwent LDLT for HCC during this period. After exclusion of patients who received preoperative LRT (*n* = 57) and those with single lesion ≤ 1 cm in size on preoperative imaging (*n* = 26), 179 patients were included in this study.

The process of donor and recipient selection, workup, and treatment allocation has been discussed previously (9, 10). The diagnosis of HCC was confirmed on a liver dynamic CT scan or MRI. Main portal vein tumor invasion and extra hepatic metastasis were major contraindications to LDLT. Patients with anticipated delay in transplantation, tumor size > 10 cm, AFP > 1,000 ng/mL, and lobar portal vein tumor invasion were routinely considered for DS.

For the purpose of this study, we staged patients based on the modified united network for organ sharing (UNOS) staging for HCC. Under this staging, MELD exception points are granted when patients have T2 HCC (single lesion ≤ 5 cm, 3 lesions ≤ 3 cm), which corresponds to Milan criteria for transplantation (2). Patients with T3 HCC are eligible for MELD exception points if successful DS to T2 HCC is demonstrated (11).

For the purpose of this study, patient demographics, tumor-related features, MELD score, AFP, and histopathological variables like grade and microvascular invasion were assessed. Median (Inter-quartile range) was reported for continuous variables, and Mann Whitney *U* test was used to determine significance. For categorical variables, chi-squared test and Fisher's test was used to determine significance. Recurrence-free survival (RFS) was calculated by subtracting date of recurrence from the date of transplantation. We used Kaplan Meier curves for survival analysis, and Log Rank test was used to determine significance. A *P*-value <0.05 was considered statistically significant. Receiver operator curve (ROC) analysis was used to determine cutoffs for continuous variables like tumor size and AFP for recurrence. All significant variables for recurrence were included in the univariate analysis. Factors with *P*-value <0.1 were included in the multivariate analysis to determine independent predictors of recurrence for patients who underwent LDLT for T1–T4 HCC. Patients with high risk factors for recurrence on multivariate analysis were excluded and thereafter RFS for T2–T4 HCC was compared. We also compared preoperative staging with histopathological staging and assessed its impact on RFS. Well-known post-transplant prognostic variables like grade and microvascular invasion (mVI) were also assessed. All analysis was performed on statistical package for social sciences (SPSS ver. 20). The study was approved by the institutional review board and ethics committee of Shifa International Hospital Islamabad (IRB # 332-1152-2020) and verbal informed consent was taken from participants.

## Results

### Patient Characteristics

**Figure 1:**
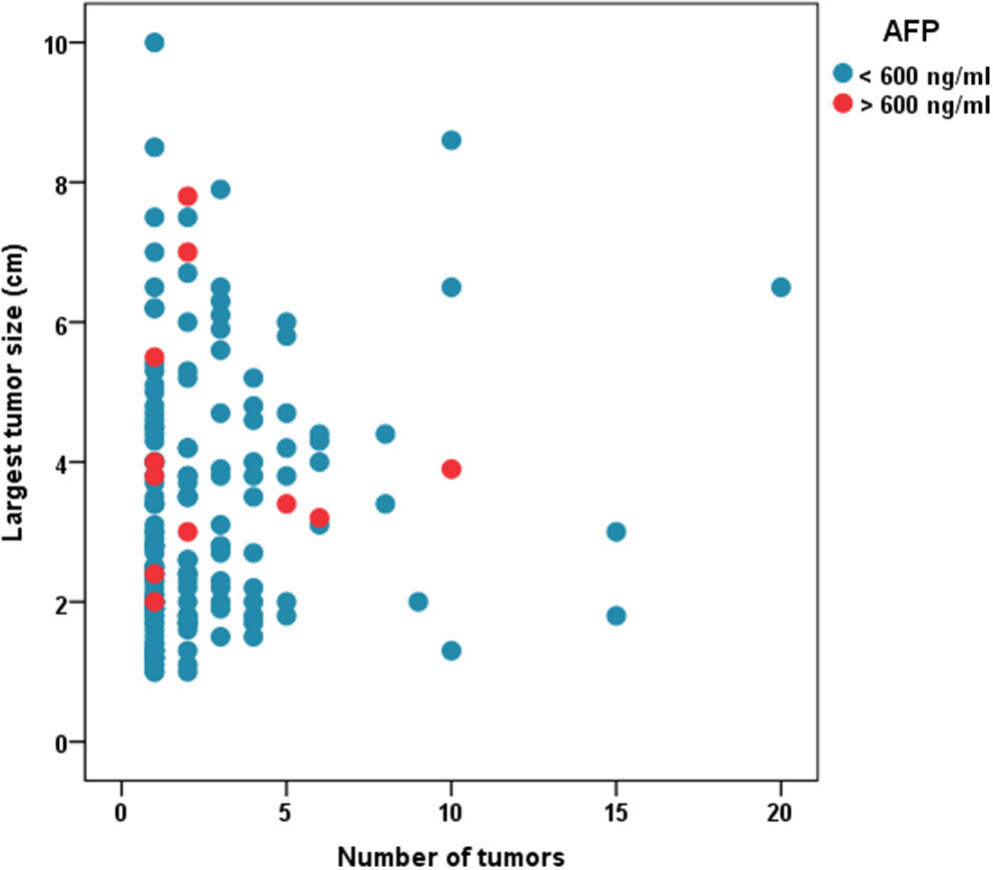
Distribution of tumor size and number in patients who underwent LDLT.

Median follow-up was 30.2(16.4–46.3) months. Figure 1 shows the distribution of patients with regards to tumor size, number and AFP cutoff of 600. Table 1 shows patient characteristics. The median AFP was 15.5 (5.3–69.2) ng/mL. The median MELD score was 19 (14–25).

**Table 1:** Patient characteristics.

		**Number(*n* = 179)**	**Percent**
Tumor size (cm)	≤5	149	83.2
	>5	30	16.8
Tumor nodules	Single	90	50.3
	≤3	143	79.9
	>3 nodules	36	20.1
Lobarity	Bilobar disease	54	30.2
MELD score	10	17	9.5
	11–20	83	46.4
	21–30	69	38.5
	31–40	10	5.6
Etiology	HCV	135	75.4
	HBV/HDV	28	15.6
	Others	16	9
Alpha fetoprotein (ng/mL)	<10	72	40.2
	11–100	66	36.9
	101–600	22	12.3
	>600	11	6.1
	Missing	5	4.5
UNOS HCC stage	T1	27	15.1
	T2	79	44.1
	T3	31	17.3
	T4a	34	19
	T4b	8	4.5
Transplant criteria	Within Milan	106	59.2
	Within UCSF	123	68.7

### Predictors of Recurrence

**Figure 2:**
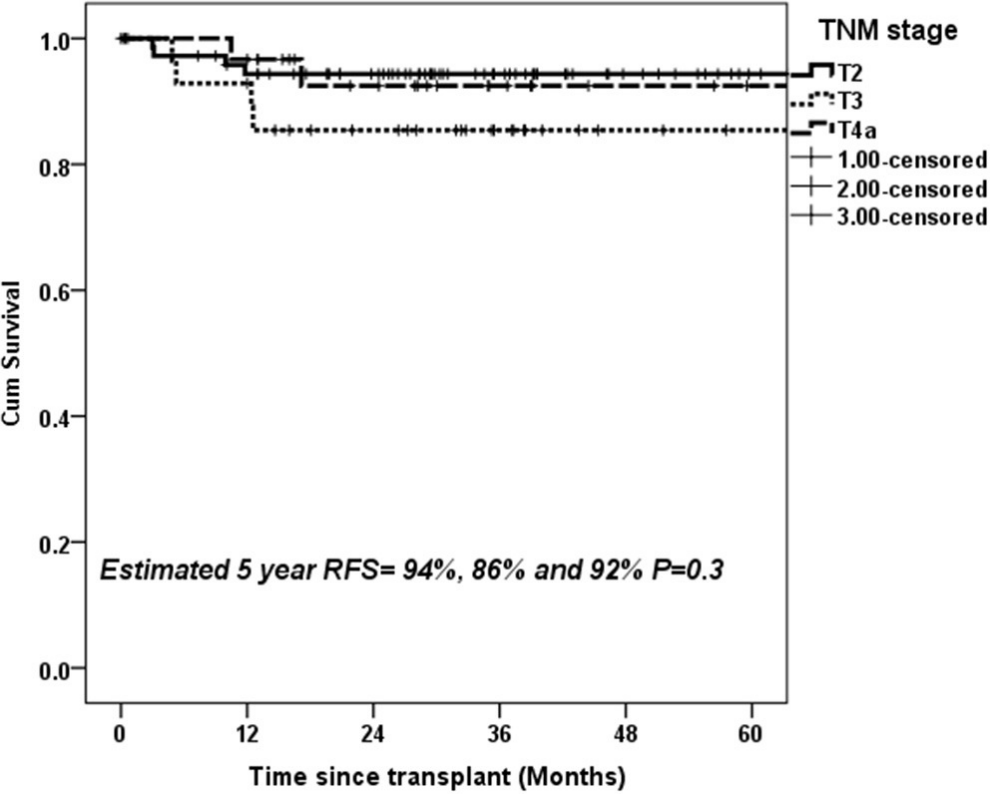
Estimated 5-year RFS with upfront transplantation in patients with AFP < 600 ng/mL and UNOS T2, T3, and T4a HCC.

On ROC analysis, tumor size cutoff of 3 cm (Area under curve = 0.64, *P* = 0.04) and AFP cutoff of 600 ng/mL (Area under curve = 0.66, *P*–value = 0.03) were significant variables for recurrence. Recurrence rate in T1, T2, T3, T4a, and T4b HCC was 0, 10.1, 16.1, 5.9, and 37.5% (*P* = 0.02) (*P* = 0.02) as shown in Table 2. On multivariate analysis, AFP > 600 ng/mL [HR:11.7, *P* < 0.001] and T4b (macrovascular invasion) [HR = 5.6, *P* = 0.03] were independent predictors of recurrence (Table 3). The 5-year RFS in patients with T2, T3, and T4a HCC with AFP < 600 ng/mL (*n* = 134) was 92.5%. The median survival was 32.2 (20.8–49.9), 32.5(16.5–39.6), and 32.4(16.1–62.9) months (*P* = 1). The 5-year RFS for T2, T3, and T4a HCC was 94, 86, and 92% (*P* = 0.3) as shown in Figure 2.

**Table 2:** Recurrence with various preoperative factors.

		**Recurrence**	**Percentage**	***P*-value**
Milan criteria	within	8/106	7.5	0.2
	outside	10/73	13.7%	
UCSF criteria	within	11/123	8.9	0.4
	Outside	7/56	12.5	
Tumor size	≤3	6/98	6.1	0.05
	>3	12/81	14.8	
Tumor number	≤3	15/143	10.5	0.7
	>3	3/36	8.3	
Lobarity	Unilobar	14/125	11.2	0.4
	Bilobar	4/54	7.4	
AFP(ng/ml)	≤600	12/168	7.1	<0.001
	>600	6/11	54.5	
TNM stage	T1	0/27	0	0.02
	T2	8/79	10.1	
	T3	5/31	16.1	
	T4a	2/34	5.9	
	T4b	3/8	37.5	
OPTN criteria for DS	T2	8/79	10.1	0.3
	DS group	5/29	17.2	

**Table 3:** Univariate and multivariate analysis for recurrence.

		**Univariate analysis**		**Multivariate analysis**	
**Prognostic factors**		**Hazard ratio (confidence interval)**	***P*****-value**	**Hazard ratio (confidence interval)**	***P*****-value**
Tumor size (cm)	<3	1 (0.8–6.1)	0.09	1 (0.3–1.5)	0.4
	>3	2.3		0.7	
AFP (ng/mL)	<600	1 (4.4–33.9)	<0.001	1 (3.8–35.4)	<0.001
	>600	12.2		11.7	
TNM	T2	1	0.05	1	
	T3	1.5 (0.4–4.6)	0.4	0.8 (0.1–3.6)	0.8
	T4a	0.5 (0.1–2.6)	0.4	0.4 (0.07–2.4)	0.3
	T4b	5.2 (1.3–19.7)	0.01	5.6 (1.1–27.4)	0.03

### Explant Factors and Recurrence

**Figure 3:**
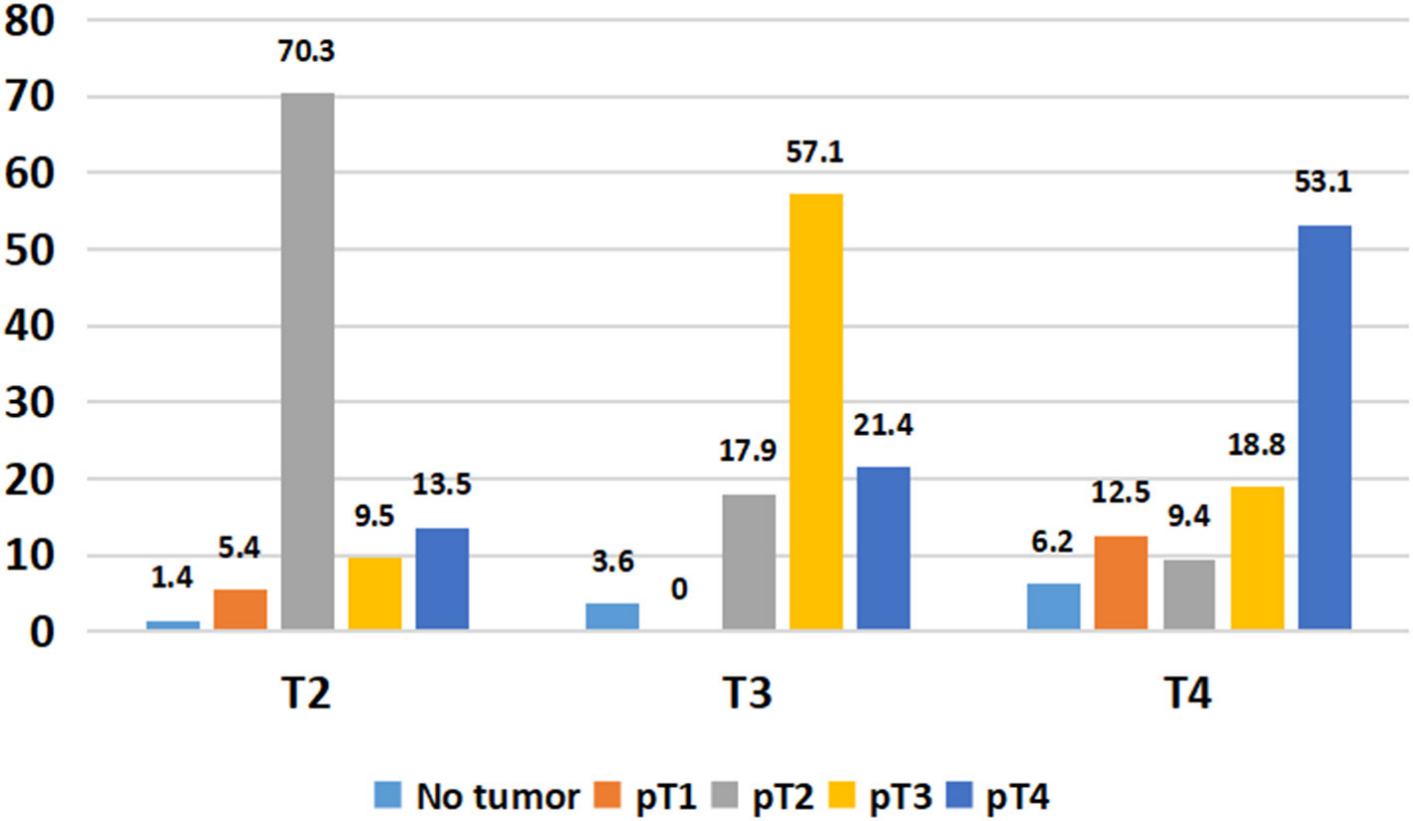
Comparison of preoperative and histopathological staging.

**Figure 4:**
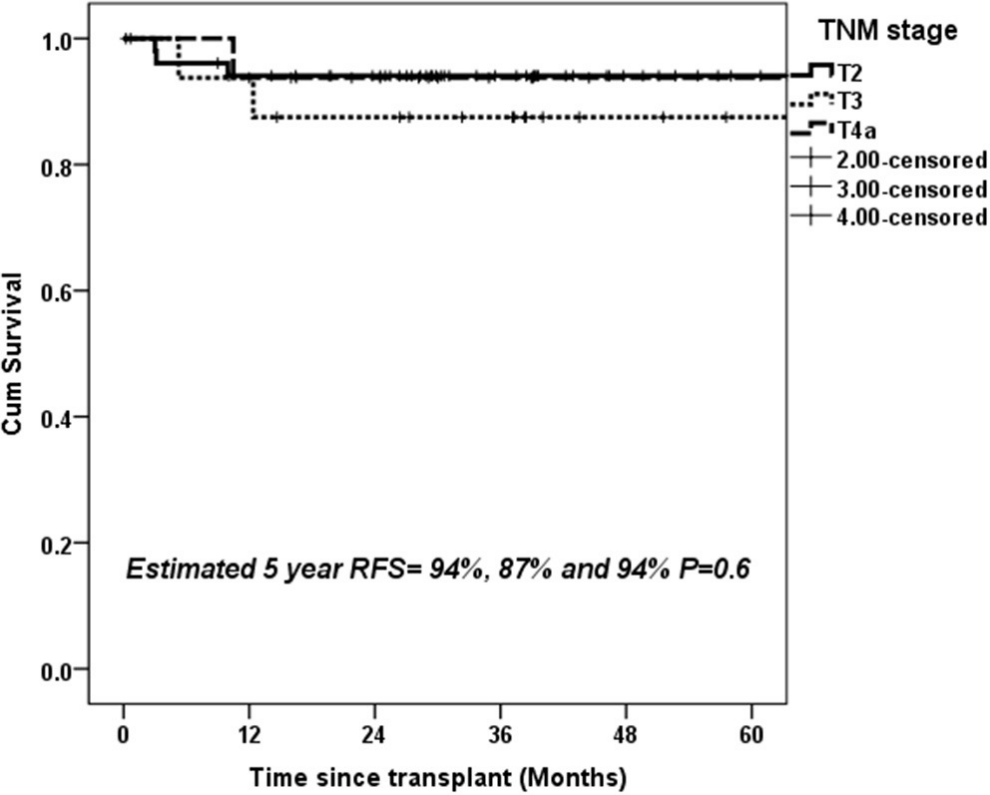
Estimated 5-year RFS with upfront transplantation in patients with AFP < 600 ng/mL and UNOS T2, T3, and T4a tumors with identical preoperative and histopathological staging.

Overall, 49/134 (36.5%) patients showed stage migration on explant histopathology. Twenty-six (19.4%) patients were overstaged and 23 (17.1%) were understaged on preoperative imaging. Preoperative staging was identical to explant staging in 70.3% with T2 HCC, 57.1% with T3 HCC, and 53.1% T4a HCC (Figure 3). Overall, 6.8% of T2 HCC, 21.5% of T3 HCC, and 46.9% of T4a HCC were overstaged on imaging (*P* < 0.001). We also looked at 5-year RFS in patients with identical T2 (*n* = 52), T3 (*n* = 16), and T4a (*n* = 17) staging on preoperative imaging and explant histopathology. The 5-year RFS in these patients was 94, 87, and 94% (*P* = 0.6) (Figure 4).

**Figure 5:**
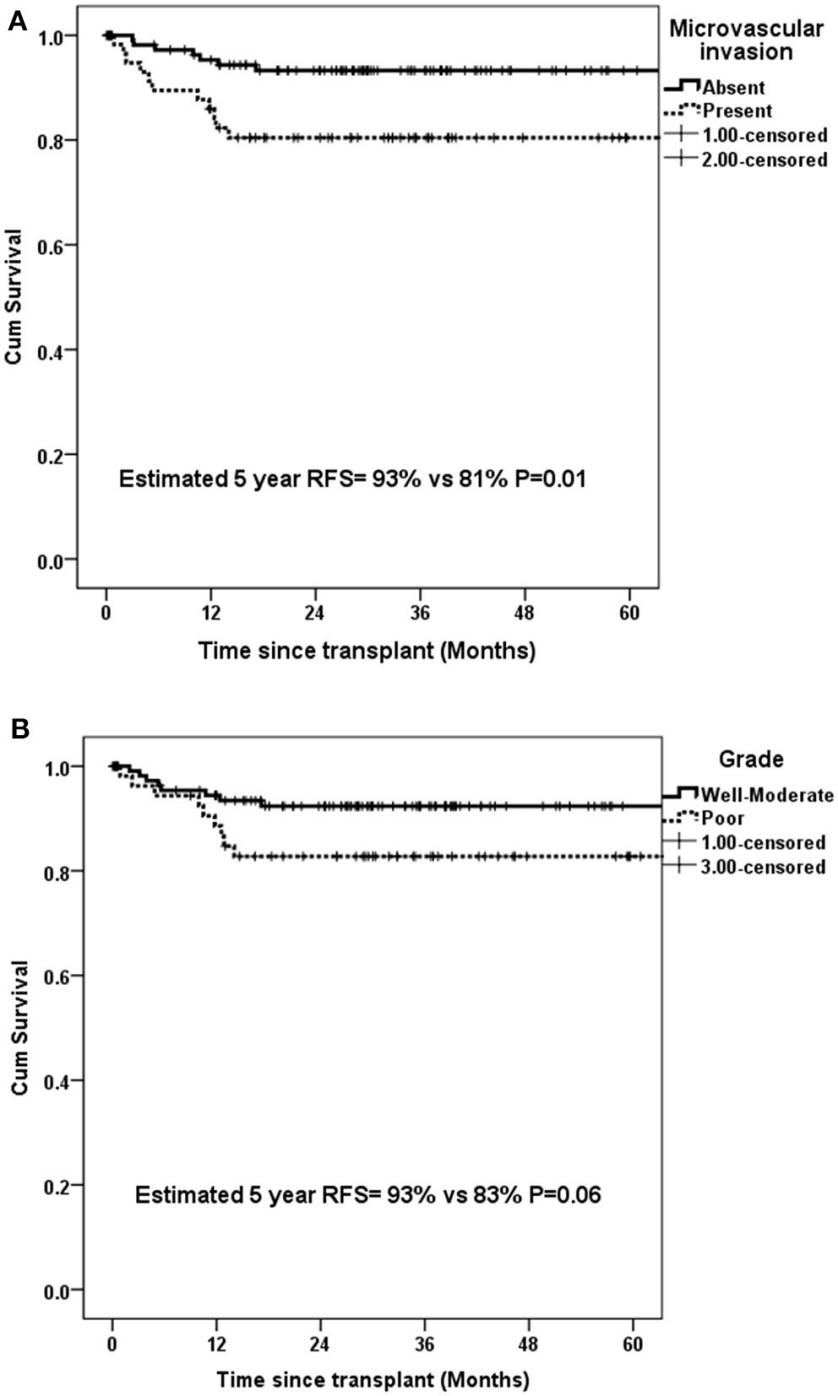
Estimated 5-year RFS in all patients with UNOS T2–T4a HCC with **(A)** microvascular invasion and **(B)** poor grade.

**Figure 6:**
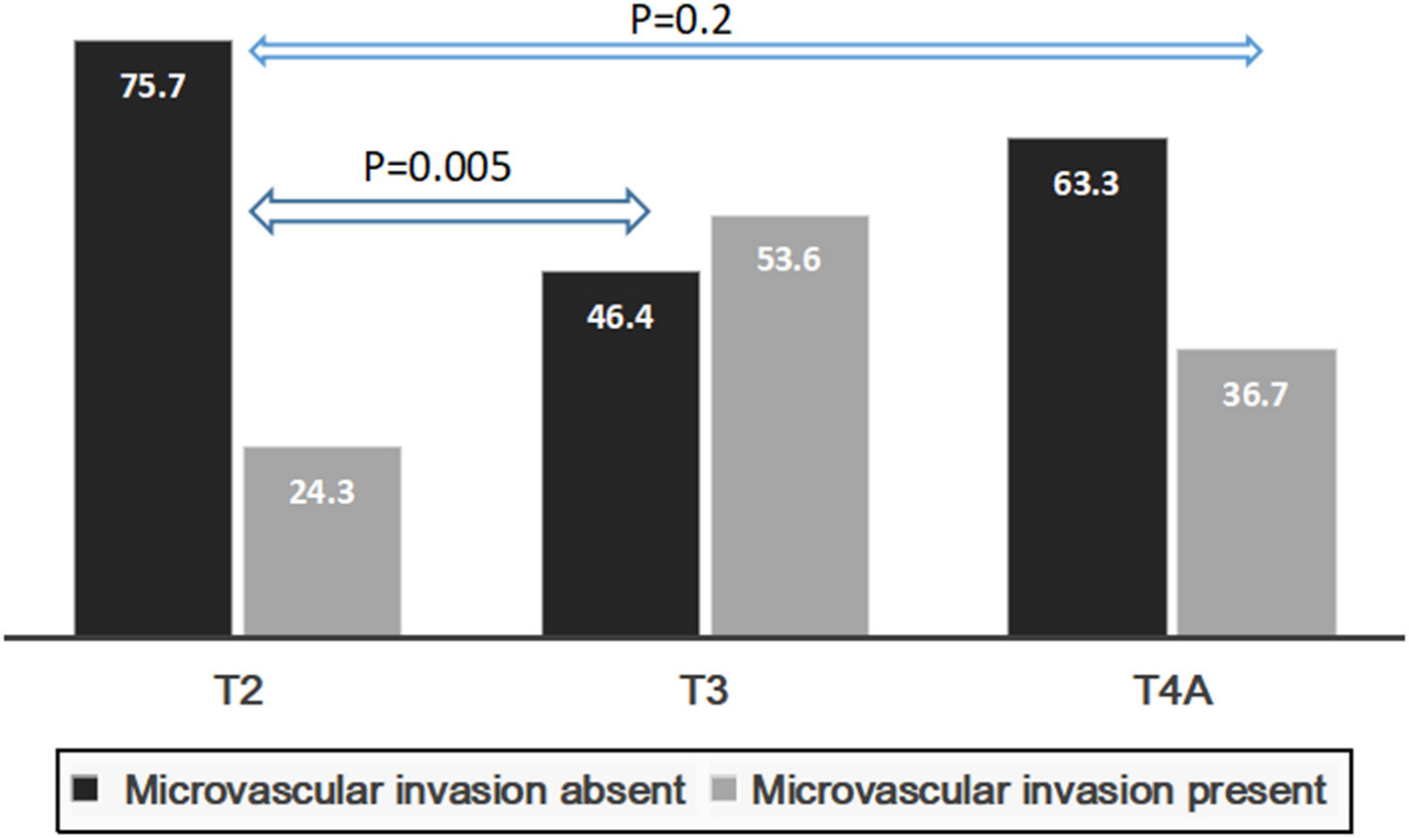
Rates of microvascular invasion in all patients with T2, T3, and T4a UNOS HCC.

Presence of mVI (*P* = 0.01) and not poor grade (*P* = 0.06) had significant impact on 5-year RFS for the whole group (*n* = 134) (Figures 5A,B). The rate of microvascular invasion was significantly different between T2 and T3 HCC (24.3 vs. 53.6%) (*P* = 0.005) but not between T2 and T4a HCC (24.3 vs. 36.7%) (*P* = 0.2), as shown in Figure 6.

## Discussion

There is increasing reliance on tumor biology for LT in HCC, and extension beyond absolute cutoffs on tumor size and numbers is being investigated. Biomarkers like AFP and prothrombin induced by vitamin K absence II (PIVKA II) are being incorporated into treatment algorithms for HCC (12). Response to preoperative LRT is a potential surrogate for tumor biology and those with favorable response have improved post-transplant outcomes (4). The current study provides evidence that upfront LT for advanced HCC (T3–T4) is a viable treatment option with comparable post-transplant outcomes in carefully selected patients (AFP < 600 ng/mL, no macrovascular on imaging).

Preoperative LRT is a suitable option for patients with well-compensated liver disease. Patients with T3–T4 HCC and decompensated liver disease or bland main portal vein thrombosis remain at high risk for post LRT deterioration (13). Moreover, there is risk of decompensation in patients with borderline liver function (14, 15). The modified UNOS criteria for HCC is based on recommendations of the American liver tumor study group (16). The latest guidelines issued by the organ procurement and transplant network (OPTN) have expanded upon previous eligibility criteria for DS and patients with 4–5 nodules, each <3 cm can be included in the DS protocol^1^ (17). It is important to recognize that the seminal reports by the Milan and UCSF group included a substantial number of patients who received LRT (2, 3). The UCSF group validated their own criteria based on preoperative imaging and a disproportionate use of pretransplant LRT with a potential risk of selection bias was observed in those exceeding Milan criteria (18). Thus, outcomes of upfront LT for advanced HCC (>T2 HCC) without prior LRT, particularly with evolving role of AFP, merit careful exploration. Our results show that patients with T2–T4 HCC have similar post-transplant outcomes when patients with AFP > 600 ng/mL and T4b tumors are excluded.

Incorrect staging based on preoperative imaging remains a major concern for treatment allocation in patients with HCC. At our center, it is mandatory to have dynamic imaging within 6 weeks of scheduled LT. Thus, time elapsed between last imaging and date of transplantation is unlikely to result in stage discrepancy. In fact, it has been shown that 20–30% of patients might be under- or overstaged at preoperative imaging when compared with histopathological staging (19–21). We noted that almost half of our T4a tumors were overstaged on preoperative imaging. Moreover, 17.9% of T3 tumors and 9.4% of T4a tumors were found to be T2 tumors on explant. These patients would have been eligible for MELD exception points in the DDLT setting and offered LT if preoperative staging was accurate. To offset the impact of inconsistency between pretransplant and explant staging on post-transplant outcomes, we did a subgroup analysis in patients with identical staging on imaging and explant, and found no significant difference in survival between T2, T3, and T4a tumors.

Poor grade and mVI are the two most important prognostic variables for HCC recurrence (2, 9, 10, 22–24). Both can only be reliably detected post operatively, and hence have little impact on decision making in the pre transplant setting. We found no statistically significant impact of poor grade but mVI on RFS in our patient cohort. Although rate of mVI was significantly different between T2 and T3 HCC, there was no difference between T2 and T4a HCC. Tumor size is a risk factor for mVI and since T4a UNOS HCC comprises of >3 tumor nodules irrespective of tumor size, this might partly explain the comparable rates of mVI and RFS between T2 and T4a HCC. However, lack of significance might also be due to relatively smaller number of patients in T4a subgroup. Although significant difference in survival was noted in patients based on mVI, those with mVI had 5-year RFS >80%. A low AFP might partly offset the negative prognostic impact of mVI in patients with HCC. It has been shown that in patients with mVI, factors like tumor size, number, and AFP level play a decisive role in eventual recurrence-free outcomes (25).

The limitations of the current study include its retrospective design. A risk of selection bias with sicker patients undergoing LT and patients with preserved liver function offered LRT cannot be excluded. This, however, is unlikely to yield a positive impact on post-transplant recurrence rates. The number of patients who underwent upfront LT with high AFP and MVI was relatively small. Worldwide, very few patients with such features undergo LT and our patient cohort represents a substantial number based on which judicious conclusions can be made. All patients in the current study underwent LDLT. Given the unique dynamics of DDLT, including waitlist times and competition for donor organs, results of the current study can only be applied to DDLT with caution. However, they do provide an opportunity to consider LDLT for such patients, particularly if LRT is also technically not feasible.

The current study demonstrates comparable post-transplant recurrence rates in patients with T2, T3, and T4a HCC based on modified UNOS criteria. Upfront transplantation is a feasible option in these patients provided that AFP is <600 ng/mL. The role of LRT as a DS modality in patients with AFP > 600 ng/mL or T4b HCC needs further assessment, perhaps in clinical trials. The results of the current study need validation in a larger patient cohort, preferably in multi center collaboration and including DDLT setting.

## Data Availability Statement

The raw data supporting the conclusions of this article will be made available by the authors, without undue reservation.

## Ethics Statement

The studies involving human participants were reviewed and approved by The IRB and Ethics Committee of Shifa International Hospital approved the study. Written informed consent for participation was not required for this study in accordance with the national legislation and the institutional requirements.

## Author Contributions

AB contributed to design, concept, analysis, manuscript writing, and review. AW contributed to data collection, analysis, and writing. NK contributed to design, manuscript writing, and review. All authors contributed to the article and approved the submitted version.

## Conflict of Interest

The authors declare that the research was conducted in the absence of any commercial or financial relationships that could be construed as a potential conflict of interest.

## Footnotes

^1^Available online at: https://optn.transplant.hrsa.gov/media/1200/optn_policies.pdf.
